# Nonalcoholic Fatty Liver Disease: Time to Take the Bull by the Horns

**DOI:** 10.5005/jp-journals-10018-1257

**Published:** 2018-05-01

**Authors:** Preetam Nath, Shivaram P Singh

**Affiliations:** 1 Department of Gastroenterology, Kalinga Institute of Medical Sciences, Kalinga Institute of Industrial Technology, Bhubaneswar, Odisha India; 2 Department of Gastroenterology, Srirama Chandra Bhanja Medical College & Hospital, Cuttack, Odisha, India

**Keywords:** Awareness, Lifestyle change, Nonalcoholic fatty liver disease, Prevention.

## Abstract

Nonalcoholic fatty liver disease (NAFLD) is the most common chronic liver disease in the world affecting almost one-fourth of the population. It may progress to nonalcoholic steatohepatitis (NASH), cirrhosis, end-stage liver disease, and liver cancer in the long run. Besides, it may make the natural history in other chronic liver diseases worse too. Furthermore, patients of NAFLD more often suffer from metabolic syndrome, ischemic heart disease, and extrahepatic malignancies than others, leading to a lower overall survival than the general population. Obesity and sedentary lifestyle are among the most important risk factors for NAFLD apart from increasing age, male sex, and certain genetic factors. Due to the rising incidence, possible adverse consequences, and the futile available treatment options, prevention is the key to tackle this health menace. Spreading awareness, adopting a healthy lifestyle with appropriate dietary modifications, regular physical activity are the cornerstones for challenging this unfolding monster.

**How to cite this article:** Nath P, Singh SP. Nonalcoholic Fatty Liver Disease: Time to Take the Bull by the Horns. Euroasian J Hepato-Gastroenterol 2018;8(1):47-51.

## INTRODUCTION

Nonalcoholic fatty liver disease refers to a hepatic condition associated with fat accumulation in the liver exceeding 5% of hepatocytes in individuals who consume little alcohol.^1^ Histologically, it encompasses a broad spectrum ranging from simple steatosis to NASH, which is virtually indistinguishable from alcoholic steatohepatitis,^2^ NASH-related cirrhosis, end-stage liver disease, and even hepatocellular carcinoma.^3^

## BURDEN

It would be euphemistic to describe the present scenario of NAFLD as an epidemic. What we really have today is a global pandemic, and we must get “Pandemic Ready” before it is too late. Various population-based studies have revealed the prevalence of NAFLD ranging from 30 to 40% in men and 15 to 20% in women in the West.^4-6^ On the contrary, the prevalence of NAFLD was found to be as high as 25% in rural predominant Indians.^7^ The global imaging-based prevalence of NAFLD too has been estimated to be around 25%.^8^ This indicates that a population of around 1.8 billion individuals worldwide have this disorder! Moreover, with gradually increasing incidence, it has become the predominant cause of chronic liver disease in most nations of the world and is expected to be the leading indication for liver transplantation in the near future.^9^ However, a matter of great concern is that the global prevalence of NAFLD is still on the rise,^10^ which mandates the attention of medical practitioners, researchers, and both national and international policymakers.

## NATURAL COURSE

There is considerable degree of uncertainty regarding the natural history and prognosis of NAFLD. This absence of uniformity is in part due to subtle individual differences in their genetics which modify their response to environmental factors and lifestyle leading to different disease phenotypes.^11^ Progression of fibrosis in patients with simple steatosis is quite uncommon; on the contrary, NASH progresses more frequently to cirrhosis, end-stage liver disease, and hepatocellular carcinoma.^5,12-14^

However, few studies suggest that simple steatosis can evolve to NASH with advanced fibrosis, implying that it may not be a completely benign condition.^14-16^ Hence, due to the potential gap between our current understanding and pathophysiology of NAFLD, we are still unable to predict which subgroup of NAFLD will worsen earlier and that too when.

## IMPLICATIONS

Long-standing NAFLD in a minority of patients can lead to NASH, and subsequently NASH-related cirrhosis, end-stage liver disease, and hepatocellular carcinoma.^12,13^ Besides, presence of NAFLD also increases the risk for fibrosis and aggravates the natural course in patients with other chronic liver diseases such as chronic hepatitis B^17^ and alcoholic liver disease.^18^ In the past, before the advent of directly acting antiviral agents, when pegylated interferon-alfa and ribavirin were the cornerstones of chronic hepatitis C therapy, presence of insulin resistance, which is an independent risk factor for NAFLD, was associated with poor sustained viral response.^19^ Besides, presence of NAFLD is frequently associated with several nonhepatic comorbidities. The most important and most frequently associated comorbidity is the metabolic syndrome or insulin resistance syndrome, and fatty liver was in fact considered as a hepatic manifestation of metabolic syndrome.^20,21^ However, recent studies favor the argument that the former is a better indicator for the latter than the existing criteria for metabolic syndrome.^22,23^ Furthermore, atherosclerotic cardiovascular diseases and extrahepatic malignancies of both gastrointestinal tract and other organs are also quite common in NAFLD, and surprisingly, they contribute more to the mortality in NAFLD than liver-related deaths.^24,25^

## RISK FACTORS

### Risk Factors for NAFLD

Among the nonmodifiable risk factors, increasing age^26^ and male sex^5^ are independent predictors. However, due to growing prevalence of obesity among younger age groups, NAFLD prevalence has increased considerably in children and adolescents.^27,28^ Besides, presence of certain endocrinological conditions like hypothyroidism^29^ and hypogonadism^30^ also predispose to NAFLD. In addition, related developments in the knowledge of genetics have identified the gene “patatin-like phospholipase domain-containing protein 3 (PNPLA3)” which is found to play a pivotal role in NAFLD.^31^

Among the modifiable risk factors, obesity is the most important risk factor according to most studies on NAFLD.^32-34^ The recent transition of nutrition in developing countries like India^35^ has led to immensely high incidence of NAFLD in the younger population,^28^ which is a matter of great concern. Abdominal obesity, which is a marker of metabolic syndrome,^36^ is currently considered as much more important than generalized obesity. Moreover, a direct association has been found between abdominal fat and liver fat.^37^ Dietary factors like higher intake of meat, fat, spicy food, and fried food render an individual more susceptible to NAFLD.^38,39^ Besides, fast food^39,40^ and soft drinks^41^ that contain artificial sweeteners, such as fructose,^42^ are also strongly associated with NAFLD. On the contrary, there are certain foods which are beneficial in NAFLD. Coffee has favorable effect on liver enzymes,^43^ fibrosis stage,^44^ hepatocellular carci-noma,^45^ and mortality in cirrhotic patients.^46^ Besides, intake of diet rich in monounsaturated fatty acids and polyunsaturated fatty acids has been found to improve hepatic steatosis.^47^ The low level of physical activity is also an important risk factor for NAFLD. The study from National Health and Nutrition Examination Survey (NHANES 2003-2004 and 2005-2006) have clearly demonstrated that NAFLD patients have low level of physical activity than the general population.^48^ Subsequently, in a retrospective study on biopsy-proven NAFLD subjects of the Nonalcoholic Steatohepatitis Clinical Research Network (NASHCRN), data have shown that there is an inverse relationship between exercise intensity and severity of liver tissue injury in NAFLD patients.^49^ This is of special significance in the context of counseling for NAFLD patients.

### Prevention of NAFLD

As for any disease, awareness is the key and essentially the first step in prevention. Surprisingly, a large proportion of the general population remains unaware of this silent yet disconcerting disease.^50^ Moreover, patient education programs are also lacking. In a recent study from Hong Kong, it was seen that 83% of the general population had never come across the term NAFLD.^51^ Another study from Italy which was conducted on general medical practitioners regarding their knowledge and practices on NAFLD revealed barely adequate awareness among the physicians, albeit significant improvements were observed following an educational interven-tion.^52^ Similarly, in a report from the USA, a significant proportion of primary care physicians were found to be unaware of guidelines on NAFLD.^53^ Expectedly, a study from coastal eastern India revealed that despite a high NAFLD prevalence, a substantial proportion of patients with NAFLD were unaware about both NAFLD and obesity, especially the harmful effects of obesity. Paradoxically, two-fifths of the obese NAFLD patients felt that they were not obese.^54^

The primary prevention of NAFLD will definitely require the elimination of the incumbent risk factors like central obesity, metabolic syndrome, and insulin resistance. The role of lifestyle modification as an effective treatment of diabetes mellitus has been shown in the Diabetes Prevention Program, which compared strict lifestyle modification (diet and exercise) with metformin and placebo in the prevention of diabetes.^55,56^ Adopting a healthier lifestyle can prevent development of NAFLD, like prevention of diabetes mellitus in high-risk groups. However, unlike diabetes, studies on prevention of NAFLD are scarce despite its high prevalence. In a study by Bae et al,^57^ it was observed that regular exercise was associated with a reduced risk of having NAFLD. Furthermore, there is an inverse relationship between degree of cardiovascular fitness and incidence of NAFLD.^58-60^ Hence, a healthy lifestyle is essential for prevention of NAFLD, and this should include:

- Regular moderate-to-vigorous exercise for at least 60 minutes on at least 5 days a week^61^
- A balanced low-calorie diet with <30% fat^62^
- Cutting down on fried food, spicy food,^39^ meat, and soft drinks^38^
- Avoidance of alcohol consumption^39,63^

Statins,^64,65^ fenofibrate,^66,67^ and probiotics^68^ have been tried as preventive strategies for NAFLD but further studies are needed to prove their role in prevention. Therefore, with no chemoprevention available, we are left with lifestyle changes as the only preventive modality for NAFLD, with awareness as the key.

It need not be overemphasized that the NAFLD menace can only be tackled by identifying the individuals at risk at a very young age and taking steps to address all the modifiable risk factors. A few small studies have demonstrated the relationship between childhood body weights with risk of adult NAFLD.^69-71^ Further, a large prospective study also demonstrated that a gain in body mass index (BMI) between the ages of 7 and 13 years was positively associated with adult NAFLD after adjustment for initial as well as attained BMI.^72^ Hence, children and adolescents should be the targets for the awareness programs on NAFLD. Education about the importance of ideal dietary composition, regular physical activities, and concept of ideal BMI (<23)^7^ should be a part of school curriculum, if we are serious about taming this silent unfolding monster.^73^

## CONCLUSION

The last couple of decades have witnessed the rise of this silent killer named NAFLD. Despite a large risk of both liver-related and cardiovascular complications, we are yet to see an ideal noninvasive tool for severity assessment as well as a safe and effective treatment. Hence, prevention is the only option to safeguard the future generation against this pandemic. Education, counseling, and lifestyle modification are the cornerstones of NAFLD prevention strategy. At the same time, we must be optimistic and put our efforts in serious research for development of an ideal treatment for NAFLD.

## References

[1] van der Poorten D, Milner KL, Hui J, Hodge A, Trenell MI, Kench JG, London R, Peduto T, Chisholm DJ, George J (2008). Visceral fat: a key mediator of steatohepatitis in metabolic liver disease. Hepatology.

[2] LaBrecque DR, Abbas Z, Anania F, Ferenci P, Khan AG, Goh KL, Hamid SS, Isakov V, Lizarzabal M, Penaranda MM, Ramos JF (2014). World Gastroenterology Organisation global guidelines: Nonalcoholic fatty liver disease and nonalcoholic steatohepatitis. J Clin Gastroenterol.

[3] Chalasani N, Younossi Z, Lavine JE, Diehl AM, Brunt EM, Cusi K, Charlton M, Sanyal AJ (2012). The diagnosis and management of non-alcoholic fatty liver disease: Practice Guideline by the American Association for the Study of Liver Diseases, American College of Gastroenterology, and the American Gastroenterological Association. Hepatology.

[4] Browning JD, Szczepaniak LS, Dobbins R, Horton JD, Cohen JC, Grundy SM, Hobbs HH (2004). Prevalence of hepatic steatosis in an urban population in the United States: impact of ethnicity. Hepatology.

[5] Vernon G, Baranova A, Younossi ZM (2011). Systematic review: the epidemiology and natural history of non-alcoholic fatty liver disease and non-alcoholic steatohepatitis in adults. Aliment Pharmacol Ther.

[6] Blachier M, Leleu H, Peck-Radosavljevic M, Valla DC, Roudot-Thoraval F (2013). The burden of liver disease in Europe: a review of available epidemiological data. J Hepatol.

[7] Singh SP, Nayak S, Swain M, Rout N, Mallik RN, Agrawal O, Meher C, Rao M (2004). Prevalence of nonalcoholic fatty liver disease in coastal eastern India: a preliminary ultrasonographic survey. Trop Gastroenterol.

[8] Younossi ZM, Koenig AB, Abdelatif D, Fazel Y, Henry L, Wymer M (2016). Global epidemiology of nonalcoholic fatty liver disease. Metaanalytic assessment of prevalence, incidence, and outcomes. Hepatology.

[9] Loomba R, Sanyal AJ (2013). The global NAFLD epidemic. Nat Rev Gastroenterol Hepatol.

[10] Zhu JZ, Dai YN, Wang YM, Zhou QY, Yu CH, Li YM (2015). Prevalence of nonalcoholic fatty liver disease and economy. Dig Dis Sci.

[11] Anstee QM, Day CP (2013). The genetics of NAFLD. Nat Rev Gastroenterol Hepatol.

[12] Adams LA, Sanderson S, Lindor KD, Angulo P (2005). The histological course of nonalcoholic fatty liver disease: a longitudinal study of 103 patients with sequential liver biopsies. Hepatology.

[13] Fassio E, Alvarez E, Dominguez N, Landeira G, Longo C (2004). Natural history of nonalcoholic steatohepatitis: a longitudinal study of repeat liver biopsies. Hepatology.

[14] Harrison SA, Torgerson S, Hayashi PH (2003). The natural history of nonalcoholic fatty liver disease: a clinical histopathological study. Am J Gastroenterol.

[15] Pais R, Charlotte F, Fedchuk L, Bedossa P, Lebray P, Poynard T, Ratziu V (2013). LIDO Study Group. A systematic review of follow-up biopsies reveals disease progression in patients with non-alcoholic fatty liver. J Hepatol.

[16] McPherson S, Hardy T, Henderson E, Burt AD, Day CP, Anstee QM (2015). Evidence of NAFLD progression from steatosis to fibrosing-steatohepatitis using paired biopsies: implications for prognosis and clinical management. J Hepatol.

[17] Bondini S, Kallman J, Wheeler A, Prakash S, Gramlich T, Jondle DM, Younossi ZM (2007). Impact of non-alcoholic fatty liver disease on chronic hepatitis B. Liver Int.

[18] Raynard B, Balian A, Fallik D, Capron F, Bedossa P, Chaput JC, Naveau S (2002). Risk factors of fibrosis in alcohol-induced liver disease. Hepatology.

[19] Romero-Gómez M, Viloria MD, Andrade RJ, Salmerón J, Diago M, Fernández-Rodríguez CM, Corpas R, Cruz M, Grande L, Vázquez L (2005). Insulin resistance impairs sustained response rate to peginterferon plus ribavirin in chronic hepatitis C patients. Gastroenterology.

[20] Uchil D, Pipalia D, Chawla M, Patel R, Maniar S, Narayani, Juneja A (2009). Non-alcoholic fatty liver disease (NAFLD)—the hepatic component of metabolic syndrome. J Assoc Phys India.

[21] Lonardo A, Ballestri S, Marchesini G, Angulo P, Loria P (2015). Nonalcoholic fatty liver disease: a precursor of the metabolic syndrome. Dig Liver Dis.

[22] Singh SP, Nath P, Singh A, Narayan J, Parida P, Padhi PK, Pati GK, Meher C, Agrawal O (2015). Nonalcoholic fatty liver disease alone is a better predictor of metabolic syndrome and insulin resistance than existing ATP-III criteria. J Metabolic Synd.

[23] Marchesini G, Bugianesi E, Forlani G, Cerrelli F, Lenzi M, Manini R, Natale S, Vanni E, Villanova N, Melchionda N (2003). Nonalcoholic fatty liver, steatohepatitis, and the metabolic syndrome. Hepatology.

[24] Vanni E, Marengo A, Mezzabotta L, Bugianesi E (2015). Systemic complications of nonalcoholic fatty liver disease: when the liver is not an innocent bystander. Semin Liver Dis.

[25] Ong JP, Pitts A, Younossi ZM (2008). Increased overall mortality and liver related mortality in non-alcoholic fatty liver disease. J Hepatol.

[26] Frith J, Day CP, Henderson E, Burt AD, Newton JL (2009). Nonalcoholic fatty liver disease in older people. Gerontology.

[27] Chhatwal J, Verma M, Riar SK (2004). Obesity among pre-adolescent and adolescents of a developing country (India). Asia Pac J Clin Nutr.

[28] Roberts EA (2007). Pediatric nonalcoholic fatty liver disease (NAFLD): a “growing” problem?. J Hepatol.

[29] Xu L, Ma H, Miao M, Li Y (2012). Impact of subclinical hypothyroidism on the development of non-alcoholic fatty liver disease: a prospective case-control study. J Hepatol.

[30] Mintziori G, Poulakos P, Tsametis C, Goulis DG (2017). Hypogonadism and non-alcoholic fatty liver disease. Minerva Endocrinol.

[31] Romeo S, Kozlitina J, Xing C, Pertsemlidis A, Cox D, Pennacchio LA, Boerwinkle E, Cohen JC, Hobbs HH (2008). Genetic variation in PNPLA3 confers susceptibility to nonalcoholic fatty liver disease. Nat Genet.

[32] Park SH, Jeon WK, Kim SH, Kim HJ, Park DI, Cho YK, Sung IK, Sohn CI, Keum DK, Kim BI (2006). Prevalence and risk factors of non-alcoholic fatty liver disease among Korean adults. J Gastroenterol Hepatol.

[33] Dassanayake AS, Kasturiratne A, Rajindrajith S, Kalubowila U, Chakrawarthi S, De Silva AP, Makaya M, Mizoue T, Kato N, Wickremasinghe AR (2009). Prevalence and risk factors for non-alcoholic fatty liver disease among adults in an urban Sri Lankan population. J Gastroenterol Hepatol.

[34] Chen CH, Huang MH, Yang JC, Nien CK, Yang CC, Yeh YH, Yueh SK (2006). Prevalence and risk factors of nonalcoholic fatty liver disease in an adult population of Taiwan: metabolic significance of nonalcoholic fatty liver disease in nonobese adults. J Clin Gastroenterol.

[35] Griffiths PL, Bentley ME (2001). The nutrition transition is underway in India. J Nutr.

[36] Carr MC, Brunzell JD (2004). Abdominal obesity and dyslipidemia in the metabolic syndrome: importance of type 2 diabetes and familial combined hyperlipidemia in coronary artery disease risk. J Clin Endocrinol Metab.

[37] Jakobsen MU, Berentzen T, Sorensen TI, Overvad K (2007). Abdominal obesity and fatty liver. Epidemiol Rev.

[38] Zelber-Sagi S, Nitzan-Kaluski D, Goldsmith R, Webb M, Blendis L, Halpern Z, Oren R (2007). Long term nutritional intake and the risk for non-alcoholic fatty liver disease (NAFLD): a population based study. J Hepatol.

[39] Singh SP, Singh A, Misra D, Misra B, Pati GK, Panigrahi MK, Kar SK, Bhuyan P, Pattnaik K, Meher C (2015). Risk factors associated with non-alcoholic fatty liver disease in Indians: a case-control study. J Clin Exp Hepatol.

[40] Charlton M, Krishnan A, Viker K, Sanderson S, Cazanave S, McConico A, Masuoko H, Gores G (2011). Fast food diet mouse: novel small animal model of NASH with ballooning, progressive fibrosis, and high physiological fidelity to the human condition. Am J Physiol Gastrointest Liver Physiol.

[41] Assy N, Nasser G, Kamayse I, Nseir W, Beniashvili Z, Djibre A, Grosovski M (2008). Soft drink consumption linked with fatty liver in the absence of traditional risk factors. Can J Gastroenterol.

[42] Ouyang X, Cirillo P, Sautin Y, McCall S, Bruchette JL, Diehl AM, Johnson RJ, Abdelmalek MF (2008). Fructose consumption as a risk factor for non-alcoholic fatty liver disease. J Hepatol.

[43] Casiglia E, Spolaore P, Ginocchio G, Ambrosio G (1993). Unexpected effects of coffee consumption on liver enzymes. Eur J Epidemiol.

[44] Modi AA, Feldman JJ, Park Y, Kleiner DE, Everhart JE, Liang TJ, Hoofnagle JH (2010). Increased coffee consumption is associated with reduced hepatic fibrosis. Hepatology.

[45] Gelatti U, Covol L, Franceschini M, Pirali F, Tagger A, Ribero M, Trevisi P, Martelli C, Nardi G, Donato F (2005). Brescia HCC Study Group. Coffee consumption reduces the risk of hepatocellular carcinoma in dependently of its aetiology: a case control study. J Hepatol.

[46] Tverdal A, Skurtveit S (2003). Coffee intake and mortality from liver cirrhosis. Ann Epidemiol.

[47] Gentile CL, Frye MA, Pagliassotti MJ (2011). Fatty acids and the endoplasmic reticulum in nonalcoholic fatty liver disease. Biofactors.

[48] Gerber L, Otgonsuren M, Mishra A, Escheik C, Birerdinc A, Stepanova M, Younossi ZM (2012). Non-alcoholic fatty liver disease (NAFLD) is associated with low level of physical activity: a population-based study. Aliment Pharmacol Ther.

[49] Kistler KD, Brunt EM, Clark JM, Diehl AM, Sallis JF, Schwimmer JB (2011). NASH CRN Research Group. Physical activity recommendations, exercise intensity, and histological severity of nonalcoholic fatty liver disease. Am J Gastroenterol.

[50] Ghevariya V, Sandar N, Patel K, Ghevariya N, Shah R, Aron J, Anand S (2014). Knowing what’s out there: awareness of nonalcoholic fatty liver disease. Front Med (Lausanne).

[51] Leung CM, Lai LSW, Wong WH, Chan KH, Luk YW, Lai JY, Yeung YW, Hui WM (2009). Non-alcoholic fatty liver disease: an expanding problem with low levels of awareness in Hong Kong. J Gastroenterol Hepatol.

[52] Grattagliano I, D’Ambrosio G, Palmieri VO, Moschetta A, Palasciano G (2008). Portincasa P; “Steatostop Project” Group. Improving nonalcoholic fatty liver disease management by general practitioners: a critical evaluation and impact of an educational training program. J Gastrointest Liver Dis.

[53] Kallman JB, Arsalla A, Park V, Dhungel S, Bhatia P, Haddad D, Wheeler A, Younossi ZM (2009). Screening for hepatitis B, C and non-alcoholic fatty liver disease: a survey of community-based physicians. Aliment Pharmacol Ther.

[54] Singh SP, Misra B, Misra D, Pati GK, Singh A, Kar SK, Panigrahi MK, Meher C, Agrawal O (2014). P841 Awareness and opinion of nonalcoholic fatty liver disease (NAFLD) patients about obesity and its consequences. J Hepatol.

[55] Tuomilehto J, Lindstrom J, Eriksson JG, Valle TT, Hamalainen H, Ilanne-Parikka P, Keinanen-Kiukaanniemi S, Laakso M, Louheranta A, Rastas M (2001). Prevention of type 2 diabetes mellitus by changes in lifestyle among subjects with impaired glucose tolerance. N Engl J Med.

[56] (2002). Diabetes Prevention Program Research Group. Reduction in the incidence of type 2 diabetes with lifestyle intervention or metformin. N Engl J Med.

[57] Bae JC, Suh S, Park SE, Rhee EJ, Park CY, Oh KW, Park SW, Kim SW, Hur KY, Kim JH (2012). Regular exercise is associated with a reduction in the risk of NAFLD and decreased liver enzymes in individuals with NAFLD independent of obesity in Korean adults. PLoS One.

[58] Perseghin G, Lattuada G, De Cobelli F, Ragogna F, Ntali G, Esposito A, Belloni E, Canu T, Terruzzi I, Scifo P (2007). Habitual physical activity is associated with intrahepatic fat content in humans. Diabetes Care.

[59] Lawlor DA, Sattar N, Smith GD, Ebrahim S (2005). The associations of physical activity and adiposity with alanine aminotransferase and gamma-glutamyltransferase. Am J Epidemiol.

[60] Zelber-Sagi S, Nitzan-Kaluski D, Goldsmith R, Webb M, Zvibel I, Goldiner I, Blendis L, Halpern Z, Oren R (2008). Role of leisure-time physical activity in nonalcoholic fatty liver disease: a population-based study. Hepatology.

[61] Dalle Grave R, Calugi S, Centis E, El Ghoch M, Marchesini G (2011). Cognitive-behavioral strategies to increase the adherence to exercise in the management of obesity. J Obesity.

[62] (2000). National Heart, Lung, and Blood Institute. The practical guide: identification, evaluation, and treatment of overweight and obesity in adults..

[63] Trovato FM, Martines GF, Brischetto D, Catalano D, Musumeci G, Trovato GM (2016). Fatty liver disease and lifestyle in youngsters: diet, food intake frequency, exercise, sleep shortage and fashion. Liver Int.

[64] Park HS, Jang JE, Ko MS, Woo SH, Kim BJ, Kim HS, Park HS, Park IS, Koh EH, Lee KU (2016). Statins increase mitochondrial and peroxisomal fatty acid oxidation in the liver and prevent non-alcoholic steatohepatitis in mice. Diabetes Metab J.

[65] Athyros VG, Boutari C, Stavropoulos K, Anagnostis P, Imprialos KP, Doumas M, Karagiannis A (2017). Statins: an underappreciated asset for the prevention and the treatment of NAFLD or NASH and the related cardiovascular risk. Curr Vasc Pharmacol.

[66] Kostapanos MS, Kei A, Elisaf MS (2013). Current role of fenofibrate in the prevention and management of non-alcoholic fatty liver disease. World J Hepatol.

[67] Shiri-Sverdlov R, Wouters K, van Gorp PJ, Gijbels MJ, Noel B, Buffat L, Staels B, Maeda N, van Bilsen M, Hofker MH (2006). Early diet-induced non-alcoholic steatohepatitis in APOE2 knock-in mice and its prevention by fibrates. J Hepatol.

[68] Brandi G, De Lorenzo S, Candela M, Pantaleo MA, Bellentani S, Tovoli F, Saccoccio G, Biasco G (2017). Microbiota, NASH, HCC and the potential role of probiotics. Carcinogenesis.

[69] Anderson EL, Howe LD, Fraser A, Callaway MP, Sattar N, Day S, Tilling K, Lawlor DA (2014). Weight trajectories through infancy and childhood and risk of non-alcoholic fatty liver disease in adolescence: the ALSPAC study. J Hepatol.

[70] Ayonrinde OT, Olynyk JK, Marsh JA, Beilin LJ, Mori TA, Oddy WH, Adams LA (2015). Childhood adiposity trajectories and risk of nonalcoholic fatty liver disease in adolescents. J Gastroenterol Hepatol.

[71] Sandboge S, Perala MM, Salonen MK, Blomstedt PA, Osmond C, Kajantie E, Barker DJ, Eriksson JG (2013). Early growth and non-alcoholic fatty liver disease in adulthood-the NAFLD liver fat score and equation applied on the Helsinki Birth Cohort Study. Ann Med.

[72] Zimmermann E, Gamborg M, Holst C, Baker JL, Sarensen TI, Berentzen TL (2015). Body mass index in school-aged children and the risk of routinely diagnosed non-alcoholic fatty liver disease in adulthood: a prospective study based on the Copenhagen School Health Records Register. BMJ Open.

[73] Singh SP (2006). Non-alcoholic fatty liver disease: the unfolding monster?. J Gastroenterol Hepatol.

